# Application of Exercised-based Pre-rehabilitation in Perioperative Period of Patients with Gastric Cancer

**DOI:** 10.1515/med-2019-0103

**Published:** 2019-11-29

**Authors:** Wu Jianjun, Wu Xing, Yao Guozhong, Zhu Chuming, Yan Jiang

**Affiliations:** 1 Liyang People’s Hospital, Liyang, 213300, China

**Keywords:** Exercised-based pre-rehabilitation, Gastric cancer, Nutritional support, Factor analysis

## Abstract

**Objective:**

To analyze the difference between exercised-based pre-rehabilitation and postoperative rapid rehabilitation of gastric cancer patients.

**Methods:**

The clinical data of 120 patients who underwent surgical treatment between 2016 and 2018 in our hospital with pathologically confirmed gastric cancer were retrospectively reviewed. According to the different treatments during the perioperative period, they were divided into exercised-based pre-rehabilitation group and postoperative rapid rehabilitation group. Factor analysis was used to analyze pre-rehabilitation and postoperative rehabilitation of patients with gastric cancer after stress response, nutritional status, insulin resistance, and inflammatory response in patients with gastric cancer, and to further evaluate the value of pre-recovery accelerated postoperative recovery.

**Results:**

The postoperative stress response, insulin resistance, and inflammatory response in the pre-rehabilitation group were lower than the conventional treatment group. The nutritional status was improved faster than the traditional treatment group. Exercised-based pre-rehabilitation for the rapid recovery of postoperative gastrointestinal function in patients with gastric cancer surgery has significant value.

**Conclusion:**

Exercised-based pre-rehabilitation has great significance for the accelerated rehabilitation of patients with gastric cancer during perioperative period.

## Introduction

Gastric cancer (GC) is the most common malignant tumors of the digestive system, which is highly malignant and can occur at any age [1]. With the improvement of living standards, the change of living habits, and the growth and aging of the population, the number of GC patients admitted to county-level medical institutions is increasing[2, 3]. Therefore, it is particularly essential to reduce the physiological and psychological traumatic stress disorder of patients after surgical procedures through a series of optimization measures during the perioperative period, so that patients can achieve early recovery.

With the introduction concept of enhanced recovery after surgery (ERAS) [4, 5, 6], the hospital stay and postoperative recovery of surgical patients have changed dramatically. More and more surgeons are paying attention to this concept. Besides, researchers emphasized that nutritional management of ERAS in various stages, preoperative treatment and postoperative rehabilitation should be closely related, and a perfect result of ERAS, which must be based on good preoperative preparation and high-quality postoperative rehabilitation. Korea researchers also suggested that dietary factors were closely associated with gastric cancer risk. They believed that a significant inverse association with gastric cancer risk was observed in flavones, even after additional adjustment for fruits and vegetable consumption in women [7].

On this basis, the concept of pre-habilitation was put forward [8, 9, 10, 11]. Recent study had found that multi-modal pre-rehabilitation programs were more effective than single-modal pre-rehabilitation programs [12, 13]. The area of pre-habilitation involved tumor surgery, sports injury, and bone injury. The interventions were similar and included home-based exercise training, nutritional counseling

and protein supplementation, and relaxation techniques administered either pre-habilitation or rehabilitation.

Currently, the exercised-based pre-rehabilitation strategies advocated that: medium and high intensity aerobic and strength exercise, protein supplement-based nutritional support, and psychological support to alleviate anxiety in the perioperative period of gastric cancer patients. However, there is no report on the application of exercise-based pre-rehabilitation in GC patients during the perioperative period. Therefore, the purpose of this study is to explore the pre-rehabilitation strategy and its practical application for GC resection patients in China and to clarify the effect of exercised-based pre-rehabilitation on perioperative functional status and prognosis of patients undergoing selective GC surgery.

## 1 Patients and methods

### 1.1 Patients

120 patients with GC confirmed by pathology and undergoing radical GC surgery in the Linyang People’s Hospital from June 2016 to June 2018. All patients were randomly divided into two groups, the experimental group (n = 60) and the control group (n = 60). The radical operation of gastric cancer in the two groups of patients was performed in accordance with the D2 standard. The patients in the group were all treated by highly qualified and experienced chief physicians. Both groups of patients were treated in accordance with the principle of controlled infusion. Two groups of patients did not have blood transfusion and blood products during surgery.

#### Patient eligibility

The inclusion criteria for enrolling patients were as follows: 1) Patients’ age were less than 75 years with GC surgery (including 75 years old); 2) No anti-cancer treatment such as radiotherapy and chemotherapy before surgery; 3) No serious cardiopulmonary insufficiency; 4) Consent of patients and their families. The exclusion criteria were listed as follows: 1) Severe organ dysfunction such as primary liver, kidney, heart, lung, and brain diseases; 2) Patients cannot undergo radical GC surgery; 3) Merge metabolic disorders; 4) Combined organectomy; 5) Patients with active dysfunction or long-term bedridden such as hemiplegia, fracture and so on; 6) Disagree with preoperative rehabilitation 7) Patients with extensive metastasis of tumors with unresectable or alleviative surgery only.

### 1.2 Control group (Postoperative rapid rehabilitation)

The implementation of basic conventional preoperative preparations such as 1) Prohibition of smoking and drinking, 2) Nutritional indicators assessment, 3) Cardiopulmonary function assessment, 4) Anesthesia risk assessment. Postoperative rapid rehabilitation was carried out. The details are as follows: 1) During surgery nursing, enhance heat insulation. 2) Encourage patients to physical exercise 3 days after surgery such as walking (at least 30min per day); 3) Dietetic instruction, to give fluid food according to the situation after surgery 6 hours.

### 1.3 Experimental group

On the basis of the conventional preoperative preparation of general surgery, the exercise-based pre-rehabilitation was carried out in a targeted manner. The details are as follows: 1) encourage patients to preoperative physical exercise and endurance training including: a) walking (at least 3000 meters per day); b) climbing (at least 8 floors per time); c) breathing (2~3 times per day for more than 10 min each time). Breathing exercises include effective coughing: patients should sit in a position and lean forward slightly, and then using abdominal muscle to take a deep breathe to cough quickly and forcefully without stopping, so as to spit out sputum. Secondly, blow balloon: patients should blow up a small balloon with strong breath and then continue to blow slowly for 5 seconds. 2) The preoperative nutritional risk score of GC patients was assessed by nutritional risk screening 2002 (NRS 2002). For patients with malnutrition risk, they should oral enteral nutrison of 0.5 barrels (provide about 1000 kal) per day before the operation. 3) Assist patients in establishing the best physical and psychological state while improving the patient’s awareness of the disease and treatment compliance. Before the experiment, the two groups of patients were included without hemiplegia, fractures, long-term bed rest, etc. There is no significant difference in mobility between the two groups. Pre-habilitation started 7 days before surgery.

### 1.4 Measures

Primary outcome: hospital stay after surgery. Secondary outcome: 1) gastrointestinal function recovery: first exhaust and defecation time, restore complete oral diet time; 2) nutritional status: body mass index, albumin, pre-albumin, hemoglobin, prognostic nutritional index (PNI); 3) insulin resistance: fasting blood sugar levels; 4) inflammatory response: C-reactive protein (CRP), white blood cell (WBC); 5) complication rate: incidence of pulmonary infection, incisional wound infection, seroperitoneum, anastomotic leakage, abdominal distention and diarrhea, arrhythmia, etc.; 6) total cost of treatment and satisfaction of patients’ families.

Wound healing is usually divided into three levels: grade A - good wound healing without inflammatory reaction , grade B - poor wound healing with inflammatory reaction, but without suppuration, grade C- healing refers to incision suppuration requiring incision drainage[14, 15, 16].

### 1.5 Statistics

Data were analyzed using SPSS version 19.0 software (IBM Corporation, Armonk, NY, USA). The measurements were expressed as mean ± standard deviation ( x̄ ± s) and the analysis between the two groups was performed *t* test for statistical calculation and to obtain *P* values. Overall, differences were considered significant at a *P* level of <0.05.

**Ethical approval**: The research related to human use has been complied with all the relevant national regulations, institutional policies and in accordance the tenets of the Helsinki Declaration, and has been approved by the authors' institutional review board or equivalent committee.

**Informed consent**: Informed consent has been obtained from all individuals included in this study.

## 2 Results

### 2.1 Patient characteristics

There were 36 males and 24 females in the experimental group (exercise-based pre-rehabilitation group), and the average age was 61.1 ± 13.5 years. While, the control group (traditional treatment group) recruited 38 males and 22 females, and the average age was 63.1 ± 13.5 years. The preoperative nutritional risk scores of GC patients were evaluated by NRS 2002, and two groups of which were all above 3. There were no significant differences between the two groups in the general characteristics of age, gender, tumor location, and clinical stage (*P* > 0.05), which was comparability.

### 2.2 Postoperative nutritional indicators of two groups

**Figure 1:**
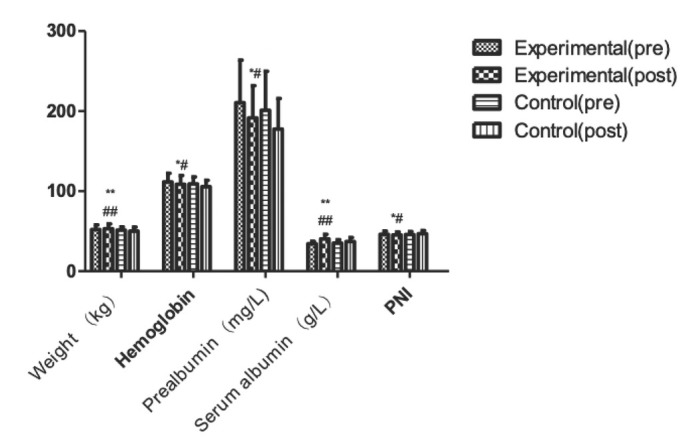
Nutritional indicators before and after treatment in two groups. Compared with control (post), *P<0.05, **P<0.01. Rate of change, Compared with control(post),*^#^P*<0.05, ^##^*P*<0.01.

There was no statistical significance of the two groups of indicators before operation. There was no statistically significant difference in preoperative nutritional indicators between the two groups (Table 1, Figure 1). However, through comparing the body weight, hemoglobin, PNI, prealbumin, and serum albumin levels of the two groups on the 7th day after operation, we discovered that the nutritional index of the experimental group was significantly higher than that of the control group (P < 0.05). We calculate the rate of change (%) of each parameter (post/ pre) in two groups.

**Table 1:** Nutritional indicators before and after treatment in two groups

Groups	Time	Weight （kg）	Hemoglobin （g/L）	Prealbumin （mg/L）	Serum albumin（g/L）	PNI
Experimental group (n=60)	Preoperative	52.1±5.7	111.6±10.7	210.7±53.2	34.3±3.1	46.1±4.1
	Postoperative	53.0±6.2	108.7±10.9	191.6±40.2	40.4±5.7	45.4±3.7

Control group (n=60)	Preoperative	51.3±4.1	109.2±8.7	201.1±48.7	35.2±4.2	45.9±3.8
	Postoperative	50.1±5.1	105.6±8.1	177.5±38.4	37.2±4.8	46.9±3.9

Rate of change (%)	Experimental	101.7±9.5	97.4±8.8	90.9±6.9	117.8±8.9	98.5±10.5
	Control	97.5±8.7	97.1±7.7	88.3±7.6	105.7±6.4	102.2±7.3

*t* value		2.798	1.768	1.965	3.326	2.161
*P-*value		0.001	0.040	0.026	0.001	0.033
*t* value-(Rate of change）		3.122	1.887	1.889	3.656	2.231
*P-*value（Rate of change）		0.001	0.039	0.031	0.001	0.031

### 2.3 Postoperative gastrointestinal symptoms and complications of two groups

Among the 60 patients in the experimental group, 4 patients developed paralytic ileus and incisional infection, respectively. Besides, there was one case of gastroparesis and one case of pulmonary infection. However, there was no deep vein thrombosis and pulmonary embolism that occurred in the experimental group. 11 patients developed paralytic ileus and incisional infection, respectively in the control group. 3 cases of gastroparesis and 3 cases of pulmonary infection in the control group. There’s a certain trend, but no statistical difference before two groups (*P>*0.05 ).

In conclusion, patients in the experimental group had less gastrointestinal symptoms and complications than the control group after surgery (Table 2).

**Table 2:** Comparison of complication rate in the two groups

Groups	Paralytic ileus	Gastroparesis	Incisional infection	Anastomotic leakage	Hypostatic pneumonia	Vascular accident
Experimental group (n=60)	2 (3.3%)	1 (1.7%)	2 (3.3%)	1 (1.7%)	1 (1.7%)	0 (0%)
Control group (n=60)	5 (8.3%)	3 (5.0%)	6 (10.0%)	3 (5.0%)	3 (5.0%)	1 (1.7%)
χ^2^	1.365	1.034	2.143	1.034	1.034	1.008
*P*-value	0.243	0.309	0.143	0.309	0.309	0.315

### 2.4 Wound healing of two groups

In the experimental group, patients with grade A, B and C wound healing were 53, 6 and 1, respectively. Compared with the experimental group, the incision healing of grade A in control group was less (Table 3, *P<*0.05). Grade B and C incision rate in the control group had an upward trend compared to experimental group, but no statistical difference (Table 3, *P>*0.05).

**Table 3:** Comparison of wound healing

Groups	Numbers n	Grade A		Grade B		Grade C	
		n	%	n	%	n	%
Experimental group	60	53	88.3%	6	10.0%	1	1.7%
Control group	60	43	71.7%	13	21.7%	4	6.7%
χ^2^		5.208		3.064		1.878	
*P*-value		0.022		0.080		0.171	

### 2.5 Time of gastrointestinal recovery and hospitalization of two groups

The time of exhaust, defecation and hospitalization in the experimental group were significantly lower than those in the control group (*P* < 0.05) (Table 4).

**Table 4:** Comparison time of gastrointestinal recovery and hospitalization

Groups	Bowel sound recovery time (h)	Exhaust time (h)	Defecation time (h)	Hospital stay (d)
Experimental group (n=60)	3.8±0.9	37.9±13.2	72.7±20.9	16.8±3.5
Control group (n=60)	4.1±0.8	43.9±13.4	90.6±22.8	18.6±4.1
*t* value	1.930	2.471	4.483	4.008
*P-*value	0.056	0.007	<0.001	0.011

### 2.6 Inflammatory indicators WBC and CRP of two groups

There was no statistically significant difference in preoperative inflammatory indicators between the two groups. However, through comparing the WBC and CRP between the two group on the 5^th^ and 7^th^ of postoperative, the inflammation level of the experimental group was lower than that of the control group.

## 3 Discussion

With the continuous improvement of the comprehensive treatment of cancer patients, the purpose of treatment for GC patients should not only be satisfied with the success of surgery, but also seek to restore gastrointestinal function, self-care ability and return to domesticity, and work as soon as possible. According to the actual situation of our hospital, this study explored the exercise-based pre-rehabilitation strategies and specific scheme of practical application for patients with GC surgery. Besides, this program is based on in-hospital management and accords with the current status of diagnosis and treatment of GC in the world. Therefore, this article mainly concerned with the basis for the development of exercise-based pre-rehabilitation strategy, the impact of exercise-based pre-rehabilitation strategy on perioperative gastrointestinal function, and the effect of exercise-based pre-rehabilitation strategy on postoperative complications. Nowadays, the exercise-based pre-rehabilitation strategy advocated high-intensity aerobic and strength exercises during the preoperative, protein supplement-based nutritional support, and psychological support to eliminate anxiety.

Recent studies have confirmed that preoperative physical exercise can promote the earlier recovery of gastrointestinal function, improve the utilization rate of nutritional support, increase anabolism, promote the absorption of inflammatory mediators, and heal wounds in cancer patients after operation. Besides, preoperative breathing exercise can significantly reduce the risk of respiratory complications in patients undergoing abdominal surgery and shorten hospital stay. What’s more, several studies showed that the mortality rate, the risk of complications, and the recovery time of gastrointestinal function are significantly increased in patients with tumors whose baseline kinetism is reduced [17, 18]. Therefore, preoperative functional exercise is very necessary for GC patients. Preoperative nutritional support can improve nutritional status, increase energy reserve for postoperative catabolism, and accelerate postoperative rehabilitation [13]. On the other hand, nutritional support can increase the benefits of exercise training [9]. Patients with scheduled surgery have a high incidence of preoperative depression of 29.7%. Meanwhile, anxiety and depression can affect the prognosis of surgery. Therefore, appropriate preoperative psychological counseling makes patients have a full understanding of the disease and prognosis and even the entire treatment process, which may play an important role in the recovery of postoperative psychological stressors.

In this study, comparing body weight, hemoglobin, PNI, prealbumin, and serum albumin levels of the two groups on the 7^th^ day after the operation, we discovered that the nutritional index of the experimental group was significantly higher than that of the control group (Table 1). Patients in the experimental group had less gastrointestinal symptoms and complications than the control group after surgery (Table 2). Compared with the experimental group, the incision healing of grade A was less, but grade B and C incision rate were higher in the control group (Table 3), which means wound healing in the experimental group were more quickly. At the same time, we compared the time of exhaust, defecation, and hospitalization in two groups, which suggested that the time of exhaust, defecation, and hospitalization in the experimental group were significantly lower than those in the control group (Table 4). Through comparing the WBC and CRP between the two groups on the 5^th^ and 7^th^ of postoperative, we found that the inflammation level of the experimental group was lower than that of the control group (Table 5). WBC and CRP have long been considered markers of inflammation. Recent studies have shown that postoperative CRP levels can predict the occurrence of incision infections and even intra-cavity infections. The value of prognosis is high, which can guide the prevention of perioperative complications. Therefore, WBC and CRP are used to reflect the effect of postoperative inflammation [19, 20]. Therefore, we believe that the appropriate preoperative pre-rehabilitation measures can effectively promote the recovery of postoperative gastrointestinal function in GC patients, reduce the physiological and psychological traumatic stress caused by surgery, and enable all aspects of patient function to reach the preoperative baseline level as soon as possible.

**Table 5:** Comparison of postoperative inflammatory indicators

Groups	Before operative		5^th^ after postoperative	7^th^ after postoperative	5^th^ after postoperative	7^th^ after postoperative
	WBC (×10^9^/L)	CRP (mg/L)	WBC (×10^9^/L)		CRP (mg/L)	
Experimental group (n=60)	6.2±0.2	2.3±0.5	7.7±1.5	7.1±1.3	12.1±5.2	9.6±4.7
Control group (n=60)	6.3±0.3	2.2±0.4	8.3±1.7	7.8±1.8	15.1±4.9	11.9±5.3
*t* value	0.1744	0.1862	2.050	2.442	3.252	2.515
*P-*value	0.861	0.853	0.044	0.016	0.001	0.007

Studies had shown that long-term bedridden of ICU patients reduced exercise will lead to a series of organ dysfunction [21, 22, 23], so long-term low exercise is an independent risk factor for cardiovascular disease. This indicates that the occurrence of postoperative complications is related to preoperative functional status. Moreover, in our study, the incidence of postoperative complications such as cardiovascular accidents, hypostatic pneumonia, deep vein thrombosis, and surgical site infection was significantly lower in the preoperative rehabilitation group than in the control group. Therefore, it shows that exercise-based pre-rehabilitation can effectively reduce the occurrence of complications after GC surgery, so as to achieve the goal of earlier rehabilitation.

In summary, this is a new study to prepare the patients before operation to better resist the traumatic stress caused by operation. Meanwhile it also restore the various functions to reach the baseline level of the body as soon as possible. Our team used an exercise-based pre-rehabilitation program to promote rapid recovery, bed turnover rate, and treatment satisfaction. Besides, it also reduced hospitalization days, labor and material consumption, postoperative complications and mortality, treatment costs, and shorten recovery time and hospital stay. Therefore exercise-based pre-rehabilitation program has important social and economic benefits. However, this study also has certain limitations. There is no unified international and domestic standard for the study of the role of exercise-based pre-rehabilitation in the perioperative period of GC patients and related evaluation indicators. It may be too early to see a definitive benefits in 5 or 7 days after a major abdominal surgery. Therefore, prolonged postoperative observation may be the most ideal method. As long as conditions permit, extending the observation time to 3 months, and ideally at least 6 months. Meanwhile, It is also possible to look for more suitable biomarkers to measure patients’ recovery. Therefore, in future studies, we will continue to explore individualized and flexible pre-rehabilitation strategies, further quantify the preoperative intervention time, and improve the intervention content and guidance methods.
